# Unique Neural Characteristics of Atypical Lateralization of Language in Healthy Individuals

**DOI:** 10.3389/fnins.2017.00525

**Published:** 2017-09-21

**Authors:** Szymon P. Biduła, Łukasz Przybylski, Mikołaj A. Pawlak, Gregory Króliczak

**Affiliations:** ^1^Action and Cognition Laboratory, Institute of Psychology, Adam Mickiewicz University in Poznań Poznan, Poland; ^2^Department of Neurology and Cerebrovascular Disorders, Poznań University of Medical Sciences Poznan, Poland

**Keywords:** language, handedness, left-handers, connectivity, specialization, laterality, resting-state fMRI, verbal fluency

## Abstract

Using functional magnetic resonance imaging (fMRI) in 63 healthy participants, including left-handed and ambidextrous individuals, we tested how atypical lateralization of language—i. e., bilateral or right hemispheric language representation—differs from the typical left-hemisphere dominance. Although regardless of their handedness, all 11 participants from the atypical group engaged classical language centers, i.e., Broca's and Wernicke's areas, the right-hemisphere components of the default mode network (DMN), including the angular gyrus and middle temporal gyrus, were also critically involved during the verbal fluency task. Importantly, activity in these regions could not be explained in terms of mirroring the typical language pattern because left-hemisphere dominant individuals did not exhibit similar significant signal modulations. Moreover, when spatial extent of language-related activity across whole brain was considered, the bilateral language organization entailed more diffuse functional processing. Finally, we detected significant differences between the typical and atypical group in the resting-state connectivity at the global and local level. These findings suggest that the atypical lateralization of language has unique features, and is not a simple mirror image of the typical left hemispheric language representation.

## Introduction

The lateralization of language is a hallmark of the brain's functional architecture. This cerebral characteristic manifests itself, for example, in that nearly 90% of right-handers use predominantly their left hemispheres during language production (Knecht et al., 2000a). Yet, a substantial number of individuals, particularly left-handers, demonstrate bilateral or even right hemispheric language representation (Knecht et al., 2000b). In accordance with some accounts, the most vivid consequences of such atypical hemispheric specialization may include various kinds of language deficits, e.g., stuttering (Fox et al., 2000). Although, much has been established about the organizational factors contributing to such deficits, very little is known about the neural underpinning of atypical lateralization of language in healthy individuals. Importantly, it is still unclear to what extent atypical language laterality is a mirror image of the typical left-hemispheric dominance.

In clinical populations, atypical, i.e., bilateral or right-hemispheric, representations of language have been linked to early injuries to the left hemispheres or epilepsy (Rasmussen and Milner, 1977). It has been widely assumed that such lesions, or even deficiencies in blood flow, particularly the ones affecting the Broca's and Wernicke's area, induce inter-hemispheric language reorganization leading to an atypical functional dominance (Lazar et al., 2000). Furthermore, on the basis of symptom similarities between children with acquired brain injuries and left-handed aphasics a hypothesis was proposed that manual preference also affects language organization, i.e., right-handedness facilitates, and left-handedness hinders the process of establishing the typical left hemispheric dominance for language (Brown and Hecaen, 1976). Finally, given the hypothesis that right hemisphere representations of functions are more diffused (Semmes, 1968), a prediction was put forward that atypical laterality should be reflected in a less focused language representation, and would occur particularly in children with an early brain injury, and in left-handers (Bishop, 2013). In sum, there is evidence from clinical populations suggesting that atypical language laterality differs from the typical representation of this function in terms of etiology and more diffused spatial distribution.

The studies investigating crossed aphasics, i.e., right-handed individuals with aphasias after right hemisphere damages, pointed, however, to quite focused contributions of the right hemispheres to language functions (Henderson, 1983; Basso et al., 1985; Alexander et al., 1989; Marien et al., 2004). Indeed, the putative representations may have even mirrored the typical organization observed in the left hemisphere, indicating that the intra-hemispheric organization of language does not depend on the side of functional dominance (Henderson, 1983). Furthermore, investigations of large groups of left-handed aphasics (Goodglass and Quadfasel, 1954; Hecaen and Sauguet, 1971) have demonstrated that in most of the cases language was lateralized typically to the left hemisphere, as in right-handers. Likewise, when right-handed aphasics were matched with left-handed aphasics with similar size and side of the lesion, education, age, length of illness, etiology, and sex, almost no differences in their aphasia profiles were found (Basso et al., 1990). Therefore, it has been suggested that a presumed link between manual preference and cerebral lateralization is in fact not mandatory (Goodglass and Quadfasel, 1954; Hecaen et al., 1981). All in all, the current interpretation of the wide range of neuropsychological evidence indicates that atypical right hemisphere language lateralization is neither diffuse nor exclusively associated with left-handedness (see also Schmitz et al., 2017), with the link to an early brain injury being doubtful, and no clear indication that it simply mirrors the one present in the left hemisphere.

With the advent of neuroimaging methods, hypotheses related to the atypical representation of language could be verified with greater anatomical precision, and thus could potentially extend or even revise our knowledge on this matter. Indeed, the patient population studies which used functional magnetic resonance imaging (fMRI) suggest that the majority of the atypical individuals exhibit a mirror image representation of the classical language centers (Staudt et al., 2002; Tivarus et al., 2012). However, there is also evidence that some individuals demonstrate intrahemispheric reorganization in the language network which manifests itself by the engagement of the left temporal lobe areas that is not observed in the typical individuals (Mbwana et al., 2009). Moreover, there is also some evidence (Voets et al., 2006) that peaks of activities within the Broca's area are not homologous. Specifically, patients with atypical laterality had the average peak of activity on the right located significantly more posteriorly than typical individuals.

Studies in healthy population support main findings from patients. Mirror-image language representations were found in right-handers in the small-scale study by Knecht et al. (2003), and not surprisingly in a large cohort of left-handed individuals by Tzourio-Mazoyer et al. (2016). There is also evidence that the atypical laterality is related to increased bilateral temporal lobe activity, an observation that suggests potential intrahemispheric reorganization (Tzourio-Mazoyer et al., 2016). Furthermore, the latter study also demonstrated that greater inter-hemispheric resting-state connectivity in homotopic regions connected via the corpus callosum is related to reduced lateralization of task-related activity (see also a review by Tzourio-Mazoyer and Seghier, 2016). These results, together with the analyses of behavioral data on atypical laterality of functions (e.g., Michałowski and Kroliczak, 2015) point to an exciting possibility that the left- and right-hemispheric language systems—despite yielding the same outputs—have in fact different connectivity with other brain networks. This issue can be approached by the analyses of location of the peaks of activity within homologous IFG subdivisions, an approach that none of the above studies adopted. If such differences exists, the right lateralized language system would not necessarily be a mirror version of its left-hemisphere counterpart, but a unique representation of this function (Price and Friston, 2002).

A support for this proposal comes from recent task-based fMRI studies which showed that a greater engagement of the Broca's area counterpart in the right hemisphere during word generation is often associated with atypical activity of regions involved in word reading (Cai et al., 2010), gesture planning (Kroliczak et al., 2011), and visuospatial processing (Cai et al., 2013). These functional regularities are also accompanied by changes in the structure of the brain, particularly in the insular cortex (Keller et al., 2011; Biduła and Kroliczak, 2015), and Heschl's gyri (Tzourio-Mazoyer et al., 2015). Based on this evidence, atypical language organization would be a consequence of substantial alterations in the structural and functional architecture of the cerebral cortex (e.g., Biduła and Kroliczak, 2015; Michałowski and Kroliczak, 2015). Such changes should be also apparent in functional connectivity, which in turn may constrain the brain's architecture (Stevens et al., 2015), leading to a very distinct pattern in individuals with atypically lateralized language.

In this way language lateralization could be seen as a factor which, depending on the initial structure of the cerebral cortex, substantially affects later structural and functional plasticity, as well as intrinsic and resting state connectivity. In particular, the influence of language laterality should be seen in the default mode network (DMN; Mazoyer et al., 2001; Raichle et al., 2001; for a recent review see also Raichle, 2015), which shares an intimate relationship with the semantic aspects of language (Binder et al., 1999). Notably, a recent study (McAvoy et al., 2016) showed that this link between default network and language lateralization could be observed even at the level of the hemispheric resting-state global signals. Therefore, it is worth to include these, often discarded, signals in the analyses of the alterations related to the language organization in heathy individuals.

In this study, we asked whether or not atypical hemispheric language organization differs from the typical representation of this function. To address this question, we tested 63 healthy individuals using fMRI. Language lateralization was measured with the verbal fluency task, and functional connectivity was assessed with the resting-state scans. Although, we found that atypical language organization mirrors, to some extent, the typical one observed in the left hemisphere, it differs substantially in the spread of cortical activity, as well as in the pattern of functional connectivity. Moreover, these results clarify the putative relationships between manual preference and language organization by showing that left-handed participants with a typical language organization did not differ from right-handed individuals who also exhibited typical, left-hemispheric language laterality. Taken together, our results provide a detailed picture of changes in the brain related to atypically organized language faculty, and suggest that such atypical asymmetry is a natural yet unique representation of function.

## Materials and methods

### Participants

Sixty-three healthy individuals (32 females/31 males; mean age ± standard deviation = 22.5 ± 3.4 years; median = 22; range = 19–39) volunteered to take part in this study. All participants were native speakers of Polish, and their handedness was assessed with the revised Edinburgh questionnaire (Oldfield, 1971): 28 of them were right-handed, 21 left-handed, and 14 were ambidextrous. Scores of +40 and above in the questionnaire indicate right-handedness, scores of −40 and below denote left-handedness, and the results in between signify ambidexterity (see Whitehouse and Bishop, 2009). A large sample of non-right-handers was on purpose included because they tend to have atypical lateralization of language (Knecht et al., 2000b; Kroliczak et al., 2011), which was a crucial premise for our study (see also Willems et al., 2014). All students who self-identify themselves as non-right-handers were encouraged by adds in the local papers, social media, and other flyers to take part in this study. After they voluntarily decided to participate, the revised Edinburgh questionnaire was administered to verify their opinions about their handedness. All non-right-handed volunteers with no contraindications for fMRI testing were included to increase heterogeneity of manual preference scores and, therefore, to omit the “restriction of range” problem in the correlation analyses. Some ambidextrals (3 participants) were truly bimanually skilled, because when they were recruited from the *Academy of Music in Poznan* (http://amuz.edu.pl/), we made sure they were proficient in playing instruments that required using both hands. None of the participants had any history of neurological or psychiatric disorders. In particular, care was taken to ensure that none of the studied individuals had any early brain injury or language impairment. Each participant provided written informed consent for voluntary participation in this study (which was a part of a greater project, e.g., Przybylski and Kroliczak, 2017), whose procedures were reviewed and approved by the Bioethics Committee at Poznań, University of Medical Sciences (Ethical Approval No. 63/12). Hence, the study methods were consistent with the principles of the 2013 World Medical Association Declaration of Helsinki (http://www.wma.net/en/30publications/10policies/b3/).

### Verbal fluency paradigm

To assess language laterality, we asked our participants to perform a cued verbal fluency task. In nearly all subjects, the experiment was carried out twice, in 2 different scanning sessions and on 2 consecutive days. This procedure was administered to increase the signal-to-noise ratio of the analyzed images. The test consisted of six 30-s task blocks, alternated with 30-s periods of rest. During task-related functional epochs, participants were required to silently generate as many words as possible beginning with a particular letter (i.e., L, M, G, K, T, or A) presented visually above the fixation cross. The letters used in this task were chosen based on the Corpus studies of the Polish language, which showed that most of the words that people spontaneously use begin with such letters.

### Resting-state paradigm

To assess functional connectivity, two resting-state scans were acquired in most of the individuals during the same sessions as the verbal fluency test. Resting-state fMRI is based upon an observation that spontaneous activity of the brain is highly structured (for a review see Murphy et al., 2013; Power et al., 2014b). Indeed, during rest distinct cerebral areas exhibit coherent signal modulations that form reproducible patterns (Damoiseaux et al., 2006), similar to those demonstrated during a specific task (Smith et al., 2009). Such connectivity patterns are constrained by the underlying anatomy (Greicius et al., 2009), yet could also be used to study polysynaptic neuronal circuits (Vincent et al., 2007).

In our study, during resting-state scans participants laid inactive for 6 min, a centrally presented fixation cross helped them not to move their eyes, they were instructed to think of nothing in particular, and not to fall asleep. The requirement of maintaining fixation was adopted because a substantial number of subjects during resting-state scans with their eyes closed drifts between wakefulness and sleep, which is likely to alter the functional connectivity (Tagliazucchi and Laufs, 2014). Moreover, it was shown that resting-state networks observed when individuals fixate on a cross are the most reliable (Patriat et al., 2013), and give the greatest effect sizes (Van Dijk et al., 2010).

### Imaging parameters

Imaging was performed at the Laboratory of Brain Imaging in the Nencki Institute of Experimental Biology with Siemens (Germany) 3 Tesla TRIO MRI scanner equipped with a 32-channel head coil. The blood oxygenation level-dependent (BOLD; Ogawa et al., 1990) T2^*^-weighted gradient echo planar images (EPI) had the following parameters: time repetition (TR) = 2,000 ms; time to echo (TE) = 30 ms; flip angle = 90°; 64 × 64 matrix; field of view (FOV) = 200 mm; 35 axial slices, 3.1-mm isotropic voxels, were acquired as a proxy to study neural responses. For detailed anatomy, in each participant, we obtained standard T1-weighted images with magnetization prepared rapid gradient echo (MP–RAGE; Mugler and Brookeman, 1990) pulse sequence: TR = 2,530 ms; TE = 3.32 ms; inversion time (TI) = 1200 ms; FA = 7°; 256 × 176 voxel matrix size; FOV = 256 mm; 176 contiguous axial slices; 1.0-mm isotropic voxels). To enhance the precision of registration between EPIs and T1-wighted images we also acquired fast spin echo SPACE (sampling perfection with application optimized contrasts using different flip angle evolution) T2-weighted structural images with the following parameters: TR = 3,200 ms; TE = 402 ms; FA = 120°; 512 × 512 voxel matrix size; FOV = 256 mm; 176 contiguous sagittal slices; 0.5-0.5-1 non-isotropic voxels. The obtained DICOM files were converted to NIFTI-1 format (http://nifti.nimh.nih.gov/nifti-1) using MRI-Convert software (http://lcni.uoregon.edu/jolinda/MRIConvert/).

### Structural imaging data analyses

Structural, i.e., T1- and T2-weighted, images were analyzed using FreeSufer v5.3 (Fischl, 2012), and FSL v5.0.6 (Jenkinson et al., 2012). First, MP–RAGE scans were averaged using FSL FLIRT (*flirt_average*) because in the vast majority of studied individuals (61) two such images were obtained. The resulting images were then processed with *recon-all* procedure available in the FreeSufer package. In short, this routine computes transformation to the Talairach atlas (Talairach and Tournoux, 1988), corrects signal inhomogeneity (Sled et al., 1998; Zheng et al., 2009), and extracts the brain (Segonne et al., 2004; Sadananthan et al., 2010). Next, non-linear atlas registration is computed, the neck is removed, and white matter is segmented. After dividing the hemispheres, the gray matter/white matter boundary is tessellated, and automated topology correction is applied (Fischl et al., 2001; Segonne et al., 2007). The obtained cortical reconstructions are then spherically registered to the fsaverage atlas (Fischl et al., 1999a,b; Buckner et al., 2004). The results of subcortical brain segmentation and overall brain size were used to test specific hypotheses related to these variables.

Skull-stripped and bias-corrected images (brain.mgz) were back projected to their native size (rawavg.mgz) and segmented into three classes, namely the gray matter, white matter, and cerebrospinal fluid using FSL FAST (Smith et al., 2004). The resulting tissue masks were then thresholded, binarized, and resampled to the resolution of resting-state EPI images. Matrix for this operation was computed using the boundary-based registration (Greve and Fischl, 2009) implemented in the FSL package (*epi_reg*).

### Verbal fluency imaging data analyses

The following preprocessing procedures were applied to the functional language data before statistical analyses: (1) non-brain tissues were removed using brain extraction tool (BET; Smith, 2002); (2) head motion during the scan was corrected with MC-FLIRT (motion correction with the FMRIB Linear Image Registration Tool; Jenkinson et al., 2002) by maximizing the correlation between each volume and the reference time-point (middle volume); (3) images were spatially smoothed using a Gaussian kernel of full width half maximum (FWHM) = 6.2 mm to reduce noise; (4) intensity of all volumes was normalized using mean-based method, and finally (5) images were temporally smoothed using high-pass filtering (σ = 50.0 s). For a given subject, each fMRI run was analyzed separately at the first level. Before statistical analyses, autocorrelation in the data was corrected using prewhitening procedure (Woolrich et al., 2001). Hemodynamic response was modeled using the double-gamma function. The single subject analyses were conducted in the native space of the studied individual. Runs from a given participant were averaged using fixed effects model implemented in FSL Feat. Intersubject analyses were, on the other hand, performed utilizing random-effects components of mixed-effects variance available with FLAME Stage 1 and 2 (Beckmann et al., 2003). These group analyses were performed in the normalized MNI-152 space (voxel size 2 × 2 × 2 mm). The resulting *Z* (Gaussianized *t*/*F*) statistic images were thresholded using *Z*-value of 3.1 and corrected for multiple comparisons using clusterwise significance threshold of *P* = 0.05 (Jezzard et al., 2003; Eklund et al., 2016). Notably, the clusterwise method of thresholding images does not set-up a minimal size or number of interconnected voxels but, instead, calculates the distribution of the largest cluster within the analyzed image, after initial thresholding at a particular *Z*-value. Based on this, a family-wise threshold is subsequently applied. Initial explanatory analysis was carried out using a more lenient, traditional threshold of Z > 2.3. Peaks of activity resulting from this investigation, specifically from a comparison of verbal activity between typical and atypical group, were used in the resting-state analyses.

Spatial normalization was performed in a series of steps using FLIRT with default cost function and interpolation method (Jenkinson and Smith, 2001). First, EPIs were aligned with T2-weighted structural images with 6 degrees of freedom (DOF). Next, T2- and T1-weighted images were registered to each other with 7 DOF. Finally, MP-RAGE scans were warped to the atlas space (Montreal Neurological Institute [MNI-152] template 2 × 2 × 2 mm) using affine transformation (12 DOF).

### Laterality measurements

Laterality index for each study participant was measured in a manner similar to Jansen et al. (2006). Specifically, a mean of 5% of voxels showing maximum activation value in one of the paired ROIs was calculated first. Then, uncorrected *Z* map was thresholded at 50% of this mean maximum activation value. Voxels that survived this thresholding were entered to the following equation: LI = [(L − R)/(L + R)] ^*^ 100, where L represents voxels that survived thresholding in the left ROI, and R denotes voxels that survived thresholding in the right ROI. A score of +100 indicates complete left hemispheric dominance, −100 complete right hemispheric dominance, and a score between 33 and −33 implies bilateral organization of language function (see Kroliczak et al., 2011).

This laterality measurement method addresses the problem of outliers and threshold dependency in a simple manner. More sophisticated procedures, such as a popular among SPM users toolbox (Wilke and Lidzba, 2007), instead of calculating the mean of 5% of voxels showing maximum activation value, use histogram analyses and the threshold problem is resolved by using a bootstrap algorithm. In an approach preferred by us (Jansen et al., 2006), thresholding at an individually adjusted level is used. Notably, we also went on and used other methods for assessing individual laterality. For example, we verified our initial participant classification based on LIs using a graph analysis (see below).

Laterality indices were measured in the Broca's area, which is one of the landmark structures of verbal fluency (Adcock et al., 2003). This area was defined by means of the probabilistic cytoarchitectonic maps implemented in the FSL package, as Brodmann areas (BA) 44 and 45 (Amunts et al., 1999). Specifically, left and right masks of each BA were thresholded at 50% of their maximum probability, added, and binarized. The left BA 44/45 mask has the size of 2,119 voxels (16,952 mm^3^), whereas the right one has the size of 1,581 voxels (12,648 mm^3^).

### Region of interest analysis

To test whether or not peaks of verbal activity within the Broca's area are located in similar anatomical locations across groups we performed a region of interest analysis. Specifically, we once again ran the average analysis for the verbal fluency test for each individual separately. However, this time this analysis was limited to the confines of the Broca's area mask used in the LI measurements. This procedure resulted in a peak of activity for each individual, which we defined as a voxel with the highest *Z*-score. Atlas coordinates of those points were compared using the *t*-test. Importantly, by using this method we were able to find a peak of activity even for a participant with bilateral representation of language. Nevertheless, as we were interested in clarifying whether atypical group has right hemispheric peaks of activity located more anterior/posterior, and/or superior/inferior to group with typical language organization, we contrasted these groups across *y*- and *z*-coordinates. One participant, who was classified as atypical—bilateral, exhibited the peak of activity in the left hemisphere. He was therefore excluded from the group comparison.

### Graph analysis of the verbal fluency neural patterns

To analyze the patterns of language lateralization among our participants we applied the procedure based on *3ddot* function from the AFNI suite. This allowed us to calculate spatial correlations between each unthresholded Z-stat image for the verbal fluency test. As before, this analysis was limited to the Broca's area defined by the same mask that we used to measure the LIs. The resulting 63 × 63 matrix entered Gephi 0.9.1 program and was turned into a graph. The matrix on which the graph is based represents Pearson spatial correlations between all voxels within the unthresholded Z-stat images from the task. The analysis was limited only to the Broca's area, namely, it was performed within the confines of the BA44/45 ROI, previously used in the LI assessment. Subsequently, the program's modular algorithm task was to detect distinct groups of neural patterns in the analyzed spatial relationships depicted by the graph (Blondel et al., 2008).

### Resting state imaging data analyses

Resting-state imaging data were analyzed using AFNI v1.8 (Cox, 1996, 2012), and FSL v5.0.6 (Jenkinson et al., 2012) packages. First, extreme values in the raw data time-series were removed (*3dDespike*), and misplacements between volumes due to between-scan head movements were corrected (*3dvolreg*). Next, MR signal intensity in the brain was normalized to a global mean of 1000 (*fslmaths*). Then linear trends were removed (*3dTcat*), and temporal bandpass filter (0.01 Hz < f < 0.1 Hz) was applied to the data time-series (*3dFourier*). Similar bandpass filter was also used to temporally smooth motion parameters obtained in the motion correction step (*1dBandpass*). After these procedures were completed, spurious variance, not related to neuronal processing, was removed by nuisance regression of the following signals: 24 motion related waveforms, signal related to the cerebrospinal fluid (and its backward difference), and time-series from the white-matter mask (also with its backward difference). Global signal was not included in this regression as its removal alters the group-level analyses qualitatively (Murphy et al., 2009), and is possibly related to the functional lateralization (McAvoy et al., 2016). In contrast, we studied hemispheric global signal as a signal of interest using the procedure described below. Finally, images were uniformly smoothed with 6.2 mm FWHM filter within the gray matter mask to reduce noise (*3dBlurToFWHM*). Note that we controlled head motion confounds using two methods, i.e., extended motion regressors (Friston et al., 1996), and uniform smoothing (Scheinost et al., 2014). No motion scrubbing was applied to the data as it disrupts temporal structure of the scan and alters the between-subjects degrees of freedom (Power et al., 2014a).

After initial preprocessing, a comparison between functional connectivity profiles of the group with typical and the atypical language lateralization was conducted. First, spherical masks of 5 mm radius centered on the coordinates of peak group differences (seed regions) from the word generation task were created (*fslmaths*). Then mean time-series were extracted from these masks (*fslmeants*), and the resting-state functional connectivity maps for each seed were calculated (*feat*) using a statistical procedure similar to the analysis of the verbal fluency activity (i.e., fixed effects were used for averaging scans in single subjects and random-effects components of mixed-effects variance were used for inter-subject analyses; *Z* > 3.1; clusterwise significance threshold of P = 0.05). Specifically, time courses of each seed, including global signal from the left and right hemisphere, were used as predictors in a multiple regression model at the individual participant level (see Hutchison et al., 2014, who used similar procedures).

Group analyses of the differences between the resting-state hemispheric global signals were performed on contrast images derived from comparing the left-to-right (left > right) or right-to-left (right > left) hemispheric signals from the initial multiple regression without any seed.

### Verification of anatomical localizations

Anatomical localizations in all analyses were verified using an atlas (Duvernoy, 1991), and probabilistic maps available in the FSL package (Eickhoff et al., 2007). Moreover, to aid sulcal and gyral identification, cortical surfaces and T1-weighted images of each individual were averaged (*make_average_subject*) to create a surface and volume representation of all study subjects' anatomy. Results of our analyses were overlaid on these averaged representations, i.e., average volume and surface. As our average study template was in correspondence with the fsaverage atlas, in which cerebral networks are included (Yeo et al., 2011), we could also identify which cerebral networks, including default mode and ventral attention systems, were altered in participants with atypical language lateralization.

## Results

First, we provide a general picture by describing the similarities and differences in brain activity of the groups with typical and atypical language lateralization. Second, we report the results of the seed- and global-based connectivity analyses, which give a more detailed description of alterations related to the atypical language organization.

### Verbal fluency task vs. rest blocks from the same test runs

**Figure 1:**
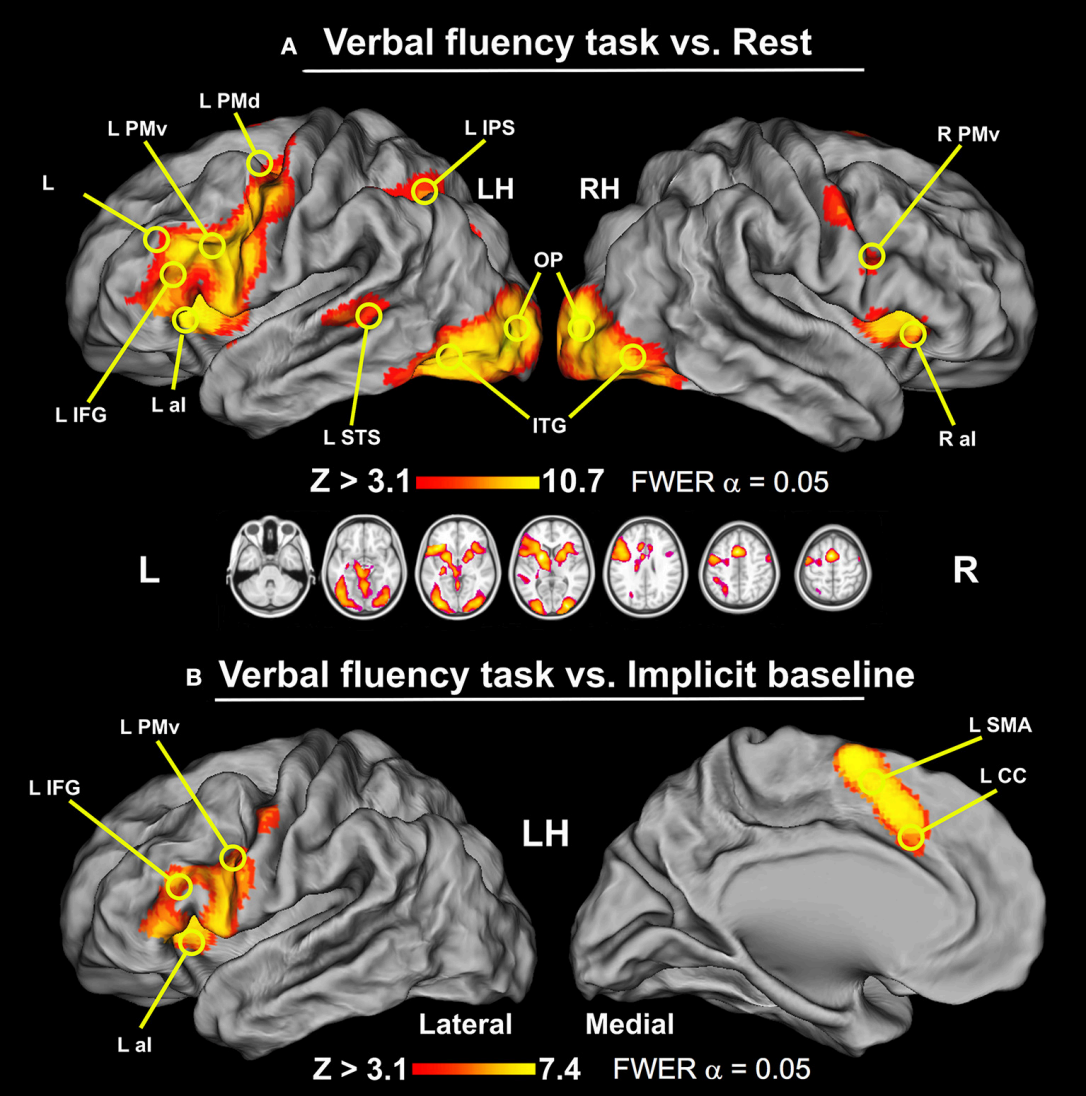
The results of whole-brain analyses for the verbal fluency task displayed on the human Population-Average, Landmark-, and Surface-based (PALS) brain atlas (Van Essen, 2005). **(A)** Verbal fluency vs. resting baseline. This contrast revealed widespread activity involving IFG, PMd, IPS, and STS exclusively in the left hemisphere, and bilateral aI, PMv, preCG, SMA complex, mCC, ITG, FG, MOG, and OP (thresholded at *Z* > 3.1, *p* = 0.05 and cluster corrected, with critical cluster size > 177 voxels). **(B)** Verbal fluency vs. implicit baseline. Two clusters of significant signal modulations were found in the left IFG and SMA complex/mCC (thresholded at *Z* > 3.1, *p* = 0.05 and cluster corrected, with critical cluster size > 182 voxels). LH, left hemisphere; RH, right hemisphere.

The brain areas activated in all participants during the verbal fluency task contrasted with the epochs of resting baseline formed widespread networks located in the frontal, parietal, temporal, and occipital cortices, particularly in the left hemisphere. In the frontal lobe, increased activity was found within the left inferior frontal gyrus (IFG), bilateral ventral premotor cortices (PMv), anterior banks of the precentral gyri, the left dorsal premotor cortex (PMd), supplementary motor area (SMA), the middle part of the cingulate cortex (mCC), and bilateral anterior insulae (aI). Notably, in the left parietal cortex we detected significant activity along and within the intraparietal sulcus (IPS). We also observed increased engagement of the left superior temporal sulcus (STS), bilateral inferior temporal gyri (ITG), and fusiform gyri (FG). Likewise, in the occipital lobe substantial activity was observed within the left and right inferior parts of the middle occipital gyri (MOG), as well as in the occipital poles (OP). There were also signal increases detected in the left and right putamen, caudate, and thalamus (Th). Finally, we observed significant signal amplifications within the anterior lobes of the cerebellum. These results are displayed in Figure 1A and the peak coordinates of identified clusters are reported in Table 1.

**Table 1:** Areas of significant signal modulations.

**Brain areas**	**MNI coordinates**			***Z*-max**	**Cluster size (voxels)**
	***x***	***y***	***z***		
**(A) Word generation vs. rest (*Z* > 8, *P* = 0.05, clusterwise corrected for multiple comparisons, reported cluster size > 100 voxels)**					
LH Putamen	−18	2	10	10.3	700
LH Supplementary Motor Area	0	2	62	10.7	513
LH Lateral Occipital Cortex	−34	−90	−4	9.6	369
LH Anterior Insula	−32	26	0	10.3	339
RH Cerebellum	32	−62	−24	10.6	313
RH Lateral Occipital Cortex	30	−92	8	10.0	288
LH Inferior Frontal Gyrus	−46	18	24	8.92	242
RH Anterior Limb of Internal Capsule	20	6	14	8.83	116
**(B) Word generation vs. implicit baseline (*Z* > 3.1, *P* = 0.05, clusterwise corrected for multiple comparisons, critical cluster size > 182 voxels)**					
LH Insula/Inferior Frontal Gyrus	−32	24	2	6.28	3,075
LH Supplementary Motor Cortex	−4	0	64	7.49	907

*LH, left hemisphere; RH, right hemisphere*.

### Verbal fluency task vs. implicit baseline

When the BOLD signal during the verbal fluency task was compared with the implicit baseline, i.e., the mean signal from the same test runs, we observed more spatially restricted effects. Specifically, this analysis revealed two circumscribed clusters of significant signal modulations in the left hemisphere. These activity patterns are shown in Figure 1B. The first set of areas included IFG, PMv, aI, and ventral parts of the precentral gyrus. The second cluster was located mainly in the vicinity of SMA.

### Laterality measurements

**Figure 2:**
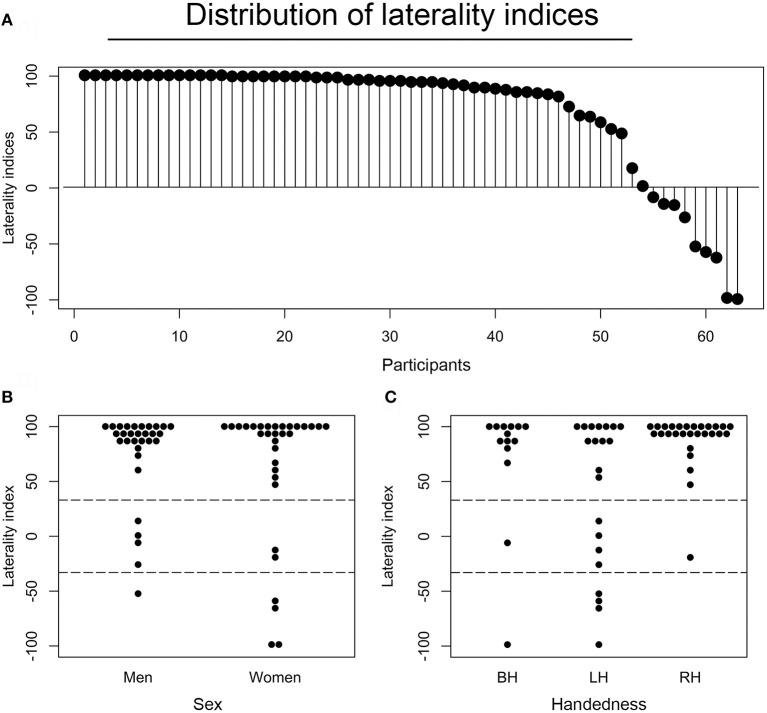
Distributions and associations of laterality indices. **(A)** Laterality indices (LIs) for each of the participants as assessed in the Broca's area (BA44/45), and presented in the descending order. Each dot represents one individual. **(B)** A distribution of LIs across sex. **(C)** A distribution of laterality indices across handedness. BH stands for both-handed (ambidextrous), LH for left-handed, and RH for right-handed individuals.

The verbal fluency activity, when contrasted with both resting and implicit baseline, showed a common cluster of lateralized signal modulation mainly in the IFG, i.e., a part of the Broca's area (Keller et al., 2009). Thus, we measured language laterality in this ROI approximated with the BA 44/45 thresholded mask. As anticipated, the vast majority of studied participants (83%) demonstrated quite typical left-hemispheric representations of language during the verbal fluency test as measured in the so-defined Broca's area. Nonetheless, a substantial number of examined individuals still demonstrated a bilateral (9%), or even right hemispheric (8%) lateralization of the studied function within this ROI. Participants that showed atypical language organization (bilateral or right hemispheric) were combined into one group for subsequent analyses. A distribution of laterality indices across all studied individuals is presented in Figure 2A.

Considering the earlier reports of the effects of age (Szaflarski et al., 2002), handedness (Knecht et al., 2000b), and sex (Shaywitz et al., 1995) on hemispheric specialization for language, we also examined the potential effects of these factors in our sample. First, there was no significant association between laterality indices and age (*r*_61_ = −0.07, *P* = 0.575), possibly due to the small age-related variability within our group (mean age 22.5 ± 3.4 years). There was, however, a significant impact of handedness on LIs. Specifically, a two (sex) by three (handedness) ANOVA revealed that right-handers were more left lateralized than left-handers (*P* = 0.005). Sex did not influence the laterality measures, as the difference between LIs for females and males was not significant (*P* = 0.224). The relevant distributions of LIs across each group of participants are shown in Figure 2B (divided by sex), and Figure 2C (divided by handedness).

### Handedness and language laterality

The above analyses showed that in our sample handedness was the only demographic variable that impacts LIs. Having the possibility to investigate this relationship further, we conducted an additional, independent-samples *t*-test to compare right- and left-handers, but only the ones with typical left-hemispheric language organization, and who participated in both fMRI sessions (and we knew their activity patterns were reproducible). This analysis showed no significant differences whatsoever between right-handers (*N* = 25, *M*_LI_ = 92.2, *SD* = 12.9) and left-handers (*N* = 12, *M*_LI_ = 88.5, *SD* = 16.9) with typically organized verbal fluency or productive language functions; *t*_(17.4)_ = –0.67, *P* = 0.511 (2-tailed; equal variances not assumed).

### Analyses and classifications of individuals in the group with atypical lateralization

As localization of language-related activity within the brain is highly variable (Ojemann et al., 1989), there are many ways in which a particular pattern of neural activity, or lack of thereof, could be classified. Moreover, neuropsychological taxonomies can be confounded, among others factors, by the syndrome drift (Kertesz and McCabe, 1977) or the influence of the subcortical lesion component (Alexander et al., 1987). Furthermore, neuroimaging methods are highly dependable on the particular threshold used in the study (Seghier, 2008). Therefore, as there is no “golden standard,” we tested our initial classification of the study participants using several complementary methods.

Neuropsychological investigations indicate that there are at least two populations of patients with atypical laterality of language (Basso et al., 1985; Alexander et al., 1989; Marien et al., 2004). The first one seems to be a mirror image of the typical representation, i.e., intrahemispheric organization of language is similar in atypical and typical group. On the other hand, the second group demonstrates atypical laterality and anomalous localization of language functions.

**Figure 3:**
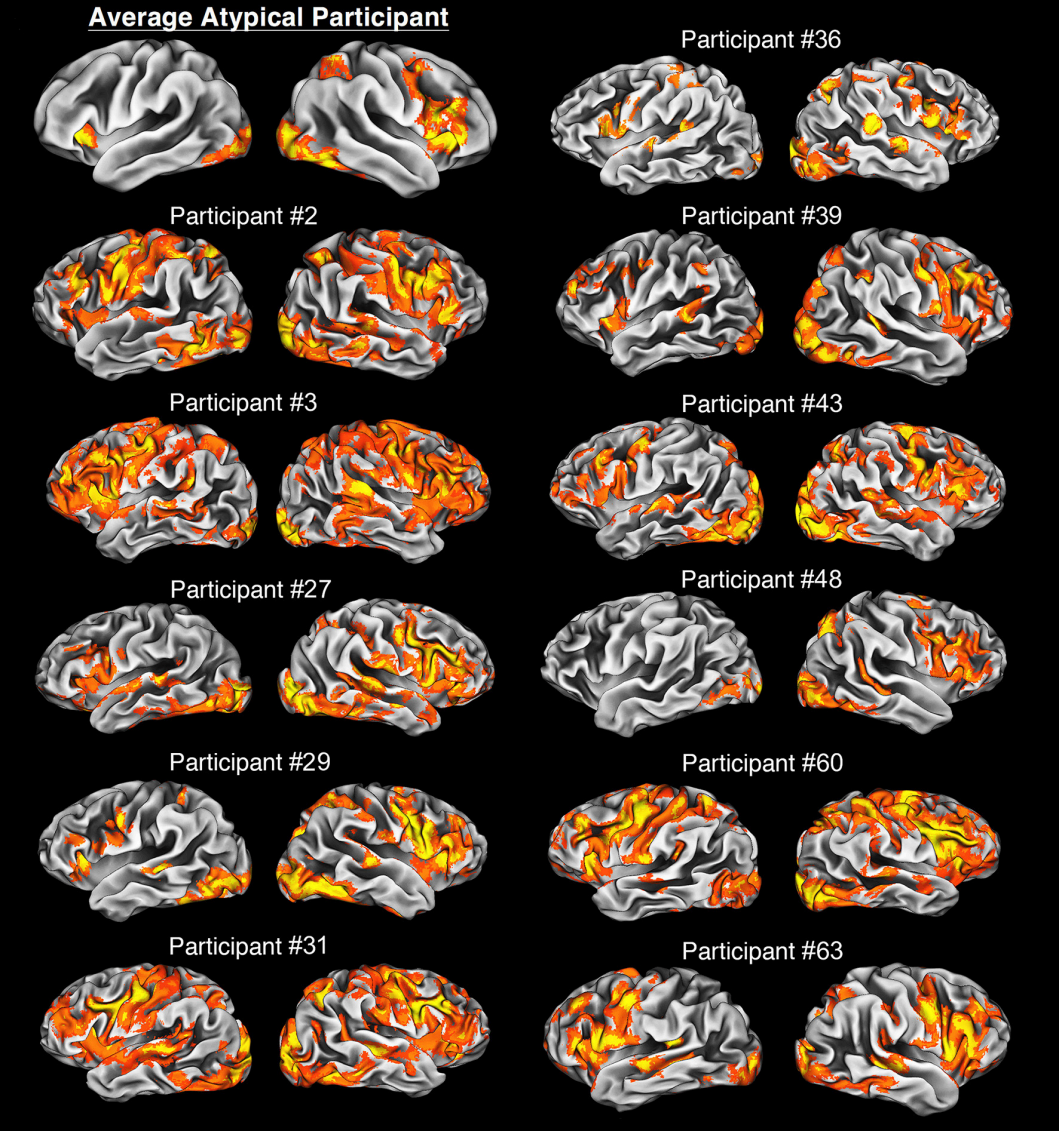
Average and individual neural activity in participants with atypical lateralization of language revealed during our verbal fluency task vs. rest. The average neural activity is overlaid on the average cortical representation of the whole atypical sample, and in all other cases, individual activity patterns are projected on individual cortical representations. All images were thresholded at *Z* > 2.3, *p* = 0.05, with clusterwise correction for multiple comparisons, and a critical cluster size adjusted for each individual. Notably, participants with the bilateral organization of language have a clear tendency to use more cortical tissue during this task, e.g., participants #2 and #3. In sharp contrast, an individual (#48) with strong right hemispheric dominance has quite focused language representation. Finally, it is of note that in general the atypical group exhibits clear right hemispheric dominance.

In our sample, all atypical individuals did engage classical language centers, i.e., Broca's and Wernicke's areas (WA) in the left and/or right hemisphere. As excepted, activity within the Broca's area was centered mainly in the posterior parts of IFG. In sharp contrast, neural activity within WA was more heterogeneous. Indeed, sometimes it exclusively engaged the superior temporal sulcus or superior temporal gyrus, but could be a mixture of activity within both of these structures. Nevertheless, none of our atypical individuals demonstrated anomalous localization of language functions, as shown in Figure 3, with the mean activity pattern displayed in the top panel on the left. Yet, due to greater variability in the localization and/or engagement of WA, this area was not revealed in the average pattern obtained for atypical representation of language.

Recently, a large neuroimaging study proposed a more refined classification of the atypical subpopulation (Berl et al., 2014). According to this report there is a three-level hierarchy of typical and atypical language patterns depending on the side, and engagement of the particular classical language areas. At the most detailed level there are 15 proposed subdivisions of individuals.

In our sample, we found that one participant clearly exhibited right hemispheric dominance with neural activity localized in the right IFG and right WA. Other individuals demonstrated less obvious patterns, with predominantly symmetrical organization, mainly with bilateral IFG and WA engagement. Nevertheless, 91% of atypical participants had peaks of their verbal activities within the Broca's area in their right hemispheres. This right-hemisphere preponderance in this group was also clearly seen in the mean activity of the sample and this activity was limited only to right IFG. Although the reasons for the lack of involvement of WA are not entirely clear, high variability in the localization of this area, among other factors mentioned above, most likely prevented us from detecting the contribution from this language center at the group level.

**Figure 4:**
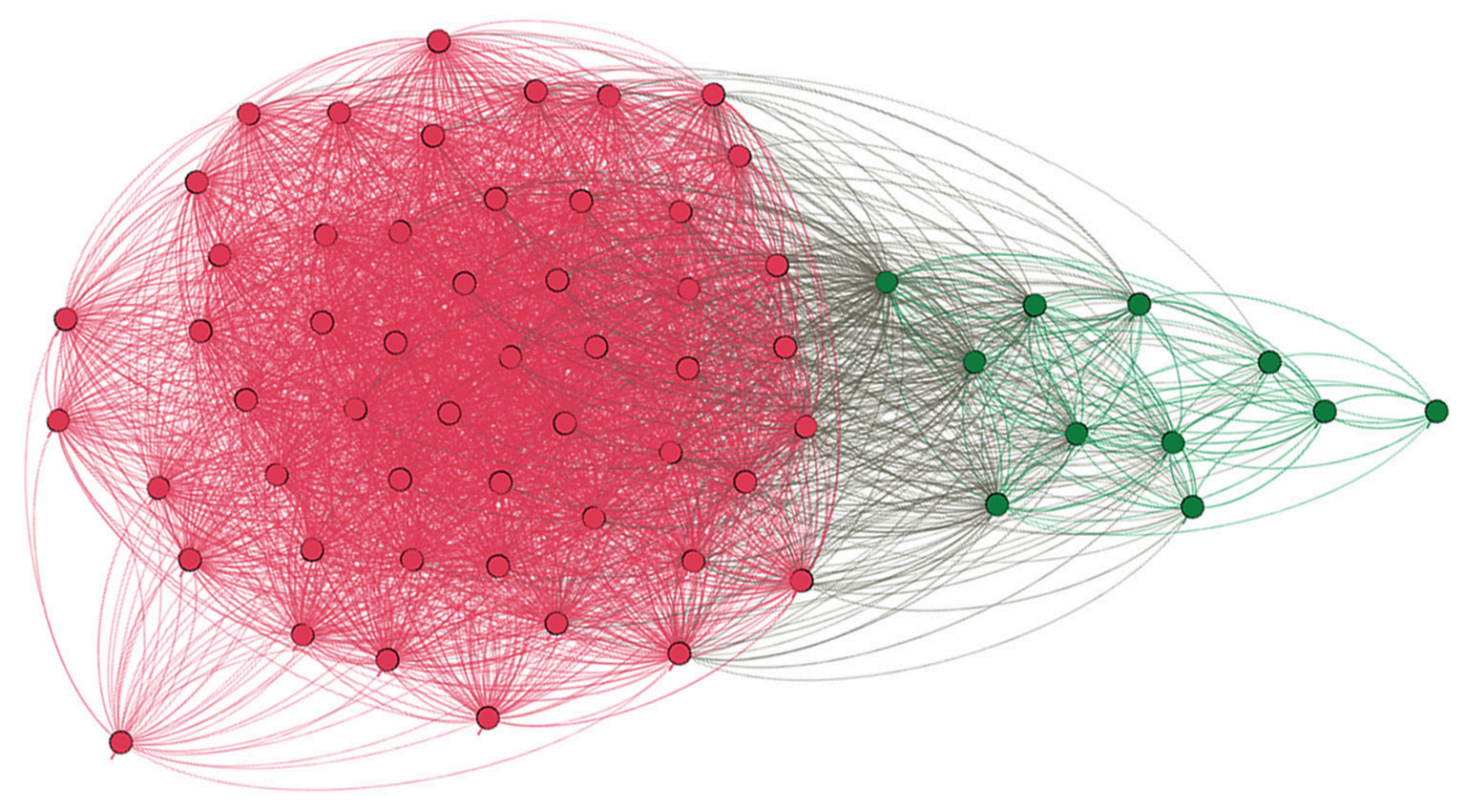
Graph depicting similarity between language patterns in the studied individuals. Each dot denotes a particular participant, each line represents similarity between language patterns as measured in the unthresholded Z-stat image within the Broca's area. Modular algorithm was used to find subgroups within all tested individuals, and the result is clear cut—two distinct groups which are depicted with different colors. Namely, magenta red represents typical participants, whereas dark green individuals with atypical laterality of language. Importantly, the number of groups and their members were exactly the same as the ones obtained using LI assessment which was based on the thresholded voxel count.

Finally, we also used a novel threshold-free classification method of pattern similarity, which divided all our participants into two groups (Blondel et al., 2008). As shown in Figure 4, the same individuals that we grouped as atypical based on the laterality measurements were also clustered together when based on significant similarity of spatial patterns of voxels engaged within the Broca's area. All in all, voxel count LI assessment, anatomical localization of activations, verification of activity peaks, and pattern similarity support our classification of the study participants into two discrete groups—typical and atypical.

### Structural analyses of participants' brains

Having the participants classified to the typical and atypical groups we formally checked whether or not there are abnormal structural differences between the studied populations. First, given a report that verbal intelligence is related to the brain volume (Witelson et al., 2006) we tested if the groups differ with regards to this variable. We found no evidence of such a difference in the brain volume (*P* = 0.43). Next, we investigated the overall cortical shape of both groups. The cortical atlases of the average typical vs. atypical participants exhibited a similar shape and similar apparent left and right asymmetries reported by other groups (Van Essen, 2005; Van Essen et al., 2012). Finally, as the caudate nucleus is thought to be a marker of structural brain abnormalities related to language (Watkins et al., 2002), we analyzed its volume in atypical and typical group and found no significant differences (*P* = 0.70 for left caudate, and *P* = 0.54 for the right counterpart).

Overall, we found no significant differences between our atypical sample and typical laterality group related to the structure of the brain. This conclusion does not preclude that there are brain structural markers related to the language lateralization. It rather means that the cortical structures of both groups are free of any apparent abnormalities that are typically related to language processing.

### Demographic differences between typical and atypical groups

We tested also if the studied groups differ in term of age and sex as these variables are thought to be related to language lateralization. We found no differences with regards to age (*P* = 0.30), or sex (*P* = 0.78).

### Common activity and its pattern for the typical and atypical group

**Figure 5:**
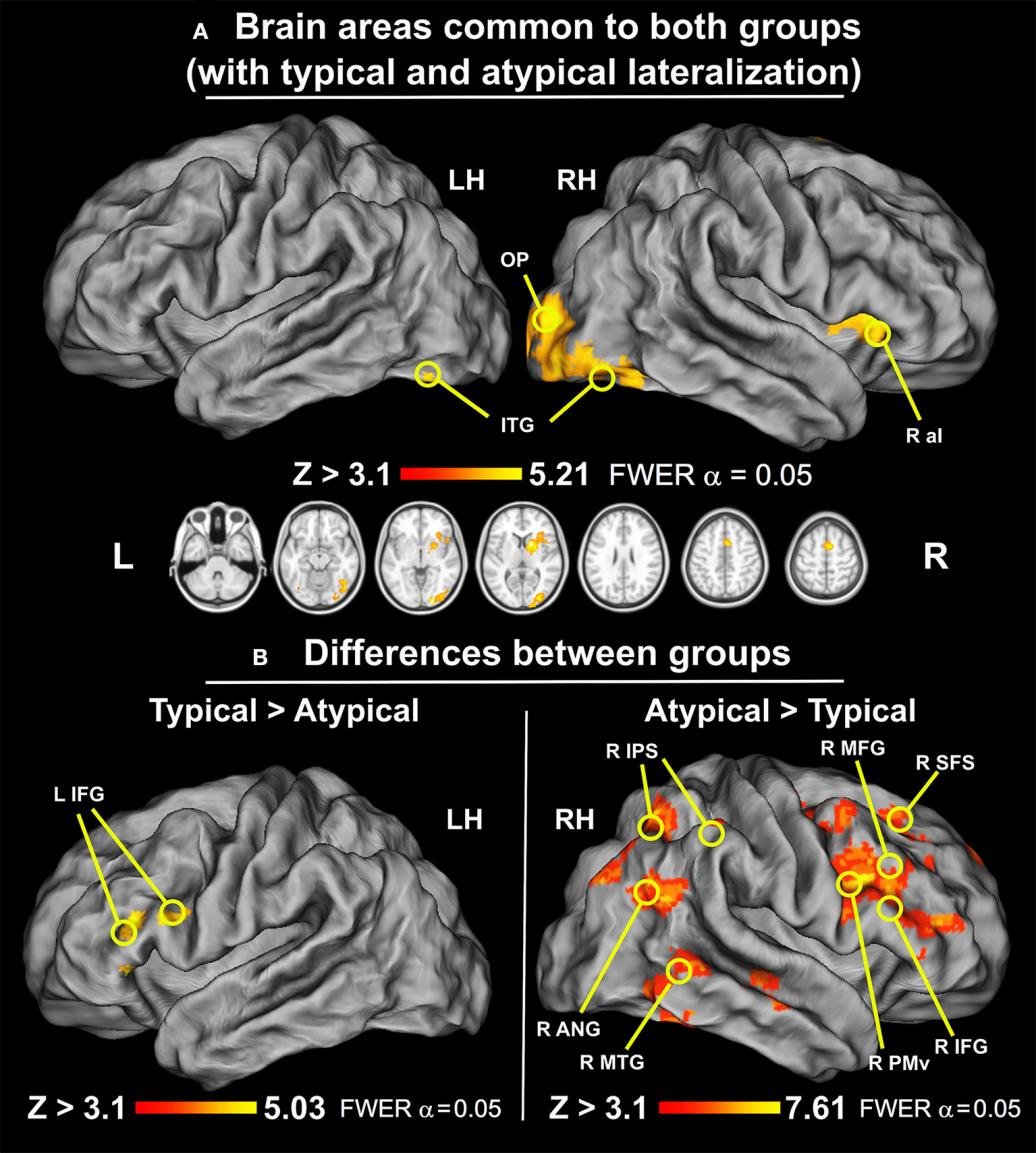
Similarities and differences between groups with typical and atypical language lateralization (thresholded at *Z* > 3.1, *p* = 0.050 cluster corrected; critical cluster size > 179 voxels). **(A)** Common regions for typical and atypical language lateralization. Both groups used right aI, MOG, FG, OP, caudate, and putamen during performance of the verbal fluency task. **(B)** Regions involved more in either typical or atypical language lateralization. Left: A direct group comparison of individuals with typical and atypical neural activity during silent word generation. Significant signal modulations were located only along the left IFG. Right: A direct group comparison of participants with atypical and typical language laterality during silent word generation. Significant modulations of neural activity were observed in right SFS, MFG, IFG, PMv, SMA, cSTG/cMTG/cITG, IPS, and ANG.

To compare the neuronal activity of a group with typical language representation and our sample with atypical language lateralization we first examined the similarity of their responses. A conjunction test with minimum statistics (Nichols et al., 2005), between the mean activity of the typical group and mean activity of the atypical sample, showed that both groups exhibited activations in aI, MOG, FG, SMA, and OP on the right during the verbal fluency task. At the subcortical level the common signal increases included primarily the right putamen, caudate, and thalamus. These results are depicted in Figure 5A, and can be interpreted as areas common to both groups regardless of the hemispheric dominance.

When the mean activation map of the sample with atypical language lateralization was flipped across the *x*-axis it was highly similar to the pattern of activity observed in typical individuals (*r* = 0.58). To further test this similarity, with the mean activity of the atypical sample flipped across the *x*-axis, we used the same conjunction test as before (Note, again, that this time the conjunction involved the flipped mean activity of the atypical sample and the regular mean activity of the typical group). This test showed that both groups engage similar areas, such as IFG and PMv, during the verbal fluency task. These areas were common to both groups, yet, of course, depend on the hemispheric laterality. Thus, it is critical to remember that they were activated in a mirror like fashion.

### Comparison of typical and atypical group during verbal fluency test

Here, we searched for group differences in the voxelwise (whole brain) analysis of neuronal activity during our version of the verbal fluency task. Such a test examines not only differences in active regions but also signal modulations in previously undetected areas.

#### Typical group > atypical group

The participants with typical representation of language showed significantly greater signal modulations within an inferior frontal cluster located in the left hemisphere. Specifically, the observed differences with the atypical group encompass the left inferior frontal gyrus through frontal operculum. This effect is shown in Figure 5B on the left.

#### Atypical group > typical group

The participants with atypical language laterality exhibited, in turn, significantly greater signal modulations in the right hemisphere, mainly in the frontal, parietal and temporal lobes. More specifically, the observed differences with the typical group encompass significant signal alterations detected in the superior frontal sulcus (SFS), MFG, IFG, PMv, and SMA. In the temporal lobe, the analysis revealed that the caudal middle temporal gyrus (cMFG) was also differentially engaged between both groups. Likewise, we found similar modulations along the IPS, and ANG. These results are depicted in Figure 5B on the right.

### Comparison of the extent of activity between the typical and atypical group during verbal fluency test

**Figure 6:**
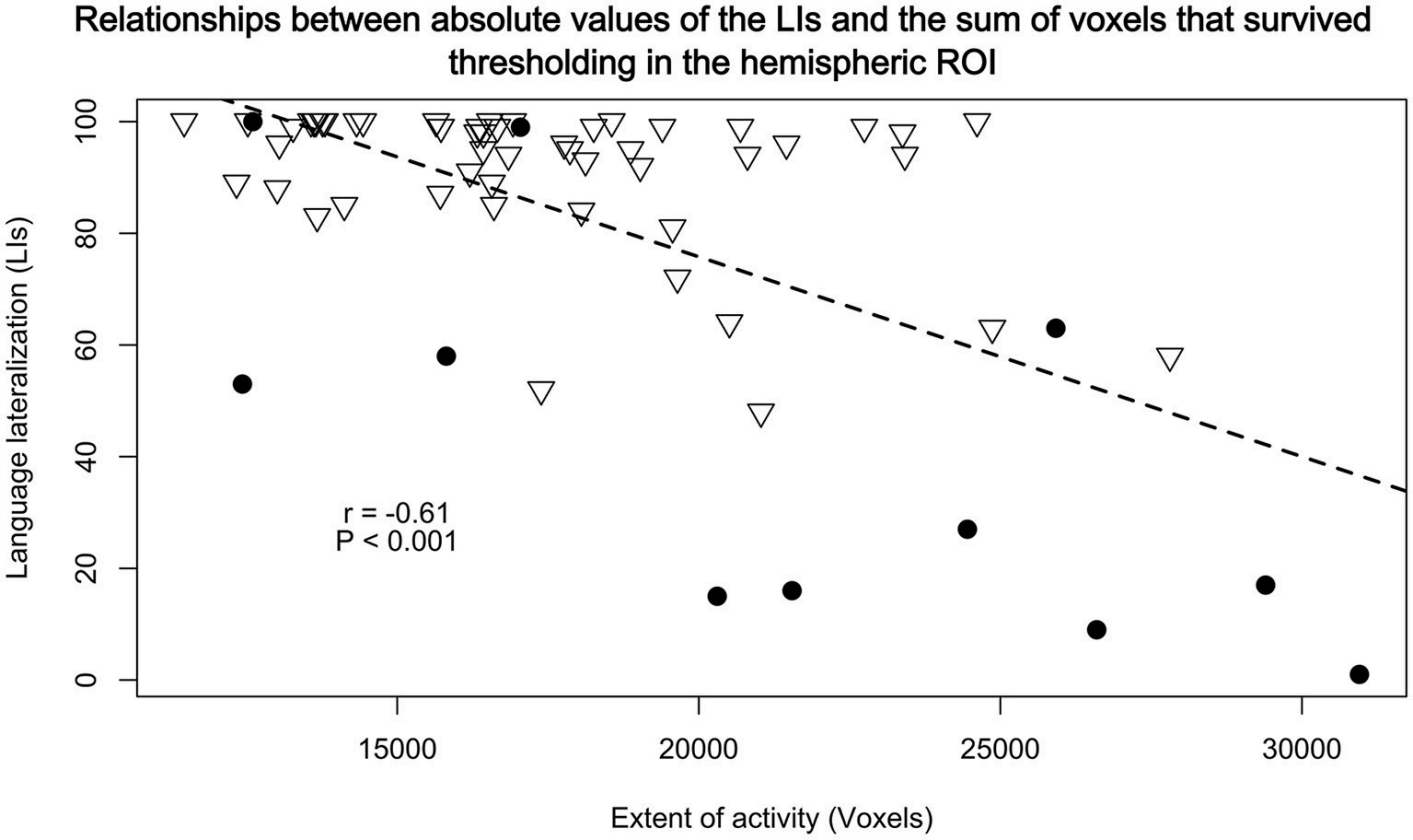
Relationships between absolute values of the LIs from BA 44/45 and the sum of voxels that survived thresholding, calculated in a similar manner as with the BA's LIs, in the hemispheric ROI. There were significant negative correlations (*r*_61_ = −0.61, *P* < 0.001) such that the less lateralized the brain activity the greater extent of language representation. Triangles represent participants with typical lateralization of language, and dots with atypical language lateralization.

Both conjunction and correlation analyses revealed that the studied participants exhibited highly similar patterns of neural responses during our verbal fluency test. However, it is still possible that the atypical language representation is more diffuse than typical organization of this function. To investigate this possibility, we first compared the relationships between the laterality indices obtained in the Broca's area and the overall number of activated voxels across the whole hemisphere. An unpaired *t*-test revealed that our sample with atypical laterality had a clear tendency to use more cortical tissue (as measured with the number voxels involved) for the control of language than our group with typically represented language [*t*_(11.35)_ = −2.07; *P* = 0.06]. There was, however, a much more interesting and significant relationship between the absolute values of laterality indices (irrespective of their direction) and the number of voxels used regardless of handedness (*r*_61_ = −0.61, *P* ≪ 0.001). The lower the laterality index (the more bilateral the activity), the more cortical tissue was involved across the whole hemisphere in the control of language. This negative correlation is depicted in Figure 6.

### Peaks of activity within Broca's area

We found a statistically significant difference between locations of peaks in the z-axis in typical and atypical group (*P* = 0.046). Specifically, atypical group had their peaks of activity located lower (mean *z* = 16) than typical group (mean *z* = 22). Counter to the previous reports of differences in the location of peaks along the y-axis (Voets et al., 2006), with atypical participants having their peaks located more posteriorly, we did not observe a significant difference along this dimension. (Although this analysis was performed on the peaks from the initial exploratory examination with traditional threshold of *Z* > 2.3, the location of peak activity should not be threshold dependent).

### A comparison of typical and atypical group during resting-state scans

**Figure 7:**
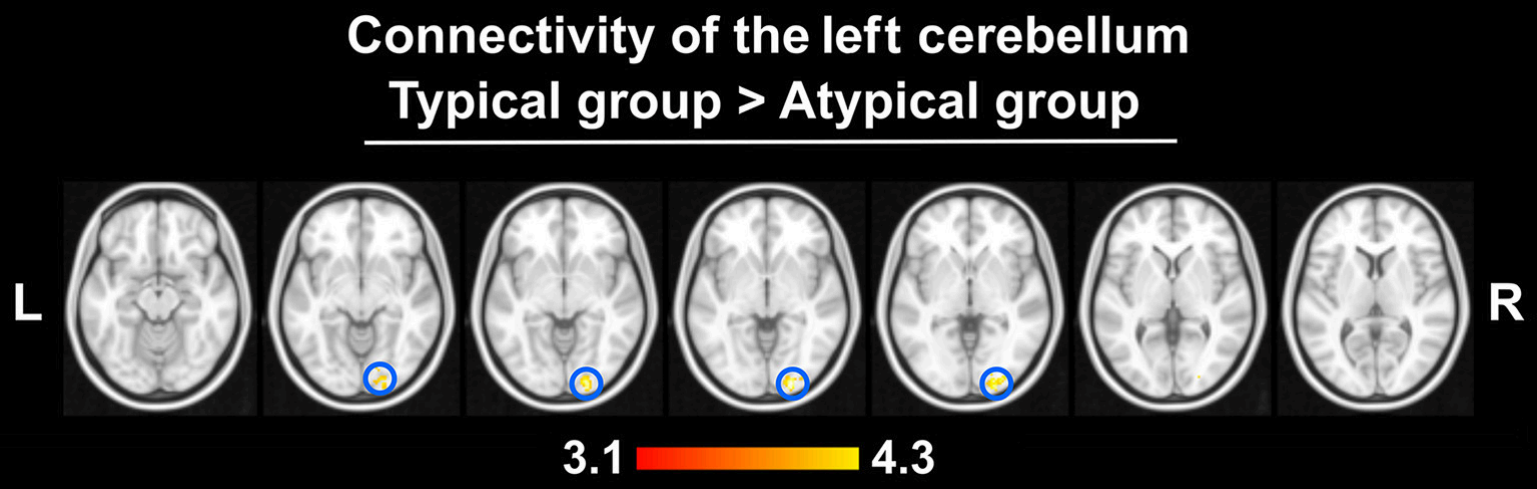
Differences in connectivity between groups (thresholded at *Z* > 3.1, *p* = 0.050 cluster corrected; critical cluster size > 69 voxels). The left cerebellum seed displayed stronger connectivity only with the right OP in the typical group. No other seeds yielded significant results.

The peak-activated voxels from the clusters enlisted in Table 2 (and found in the previous group comparisons) were used as seeds for the connectivity analyses. While virtually no differences in connectivity patterns were detected, this analysis revealed that the left-hemisphere cerebellar seed exhibited significantly stronger connectivity with the right OP in the typically lateralized group. These results are presented in Figure 7.

**Table 2:** Areas of significant group differences from initial exploratory analyses (*Z* > 2.3, *P* = 0.05, clusterwise corrected for multiple comparisons, critical cluster size > 667 voxels).

**Brain areas**	**MNI coordinates**			***Z*-max**	**Cluster size (voxels)**
	***x***	***y***	***z***		
**(A) Typical group > Atypical group**					
LH Inferior Frontal Gyrus	−42	30	0	3.64	689
**(B) Atypical group** > **Typical group**					
RH Middle Frontal Gyrus	40	16	26	4.51	6,772
RH Intraparietal Sulcus	34	−58	52	3.99	3,305
RH Inferior Temporal Gyrus	52	−46	−18	3.88	2,687
LH Cerebellum	−24	−86	−22	3.9	1,289

*RH, right hemisphere; LH, left hemisphere*.

### Comparison of the resting-state global signal between typical and atypical group

**Figure 8:**
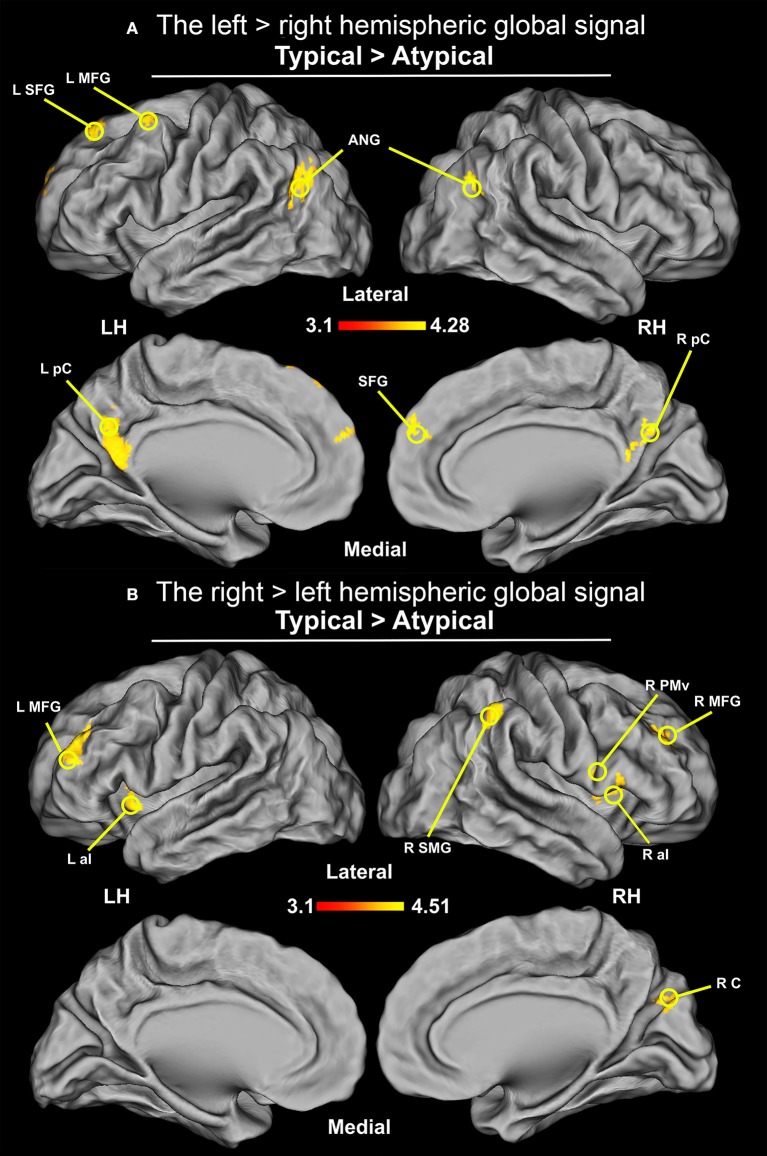
Differences in the distribution of the hemispheric global signal between groups (thresholded at *Z* > 3.1, *p* = 0.05 cluster corrected; critical cluster size > 71 voxels). The signals come from the initial multiple regression performed without any seed. **(A)** Distribution of global signal from the between-groups comparison of the left hemisphere contrasted with the right hemisphere. Increased connectivity was observed in ANG, pC, and SFG, as well as MFG for the group with typical language laterality. **(B)** Distribution of global signal from the between-groups comparison of the right hemisphere contrasted with the left hemisphere. Increased connectivity was observed in SMG, PMv, and cuneus (RC)/parieto-occipital sulcus, as well as in aI, and rostral division of MFG for the typical language laterality group.

Given a recent report (McAvoy et al., 2016) which suggests that lateralization is also reflected at the level of the brain's global signal, i.e., mean signal across the whole brain during a resting-state scan, we also examined whether or not there are asymmetries in the hemispheric global signal distribution between the studied groups. As anticipated, the global signal from the left hemisphere, contrasted with the right-hemisphere signal, in typical group was greater in the ANG, precuneus (pC), SFG, and left caudal MFG. Using the network atlas available (Yeo et al., 2011), in the fsaverage template we were able to identify the above mentioned regions as a part of the DMN. In contrast, the right hemisphere signal, when compared to the left hemisphere signal, was greater in the typical group in the supramarginal gyrus (SMG), aI, aMFG, and right cuneus. These regions, when compared with the fsaverage network template, were shown to be a part of the ventral attention network. The opposite pattern, i.e., right hemisphere signal dominance in the ANG, pC, SFG, and left caudal MFG; and greater left hemisphere signal in SMG, aI, aMFG have been observed for our sample with atypical language laterality. The results of the comparisons between the typical and atypical group are displayed in Figure 8.

## Discussion

This study investigated the neural underpinnings of atypical lateralization of language in healthy individuals and examined the idea that atypical laterality is a mirror image of the left hemispheric language dominance. We found that both studied groups used similar brain structures in a mirror fashion during our verbal fluency task. However, atypical sample also engaged the right hemispheric DMN components. Moreover, we found that atypical organization of language entails more diffuse processing and/or mechanism. Finally, we detected alterations in the resting-state intrinsic connectivity at the local and global level. These findings shed a new light on our understanding of the hemispheric differences in the organization of language in three key ways.

First, we have found that atypical laterality is associated with substantially greater engagement of frontal and temporal structures in the right hemisphere. Such differences cannot be explained in terms of mirrored representation because individuals with typical lateralization did not show a reversed pattern. Thus, atypical representation of language entails qualitative differences in its general organization. Second, although we have demonstrated that both groups exhibited highly similar overall pattern of functional activity, a more diffuse—i.e., wider in its extent—representation of language was clearly associated with its bilateral organization. Thus, atypical laterality also entails sharp quantitative differences in the representation of language skills. Third, we have shown that the connectivity patterns of the cerebellum get altered—are substantially weaker—in the atypical language representation. Finally, there are also clear differences between the two groups in the spontaneous activity/connectivity patterns revealed by the distribution of the hemispheric global signal from resting-state scans. These effects were particularly pronounced in the ventral attention and DMNs in the atypical, as compared to typical, language laterality. The unique quantitative and qualitative differences in neural processing, associated in this study with atypical lateralization of language at several levels of analysis, provide strong and convincing pieces of evidence that atypical lateralization is not a simple mirror image of the typical left hemispheric language specialization.

### Atypical lateralization of language: abnormality?

It has been suggested for a long time that atypical lateralization of language results from an early brain injury. Nonetheless, we have found bilateral or right hemispheric language representation in 17% of 63 healthy participants examined in the present study. It should be emphasized that none of these individuals had any sign of structural brain abnormalities or any obvious language impairment, which could cause or be linked to atypical laterality. Specifically, overall brain volumes, cortical shapes, and the sizes of the caudate nuclei, with the latter (when smaller) being considered critical markers of language impairments in the brain (Watkins et al., 2002), did not differ between groups. These observations are consistent with the outcomes from previous reports in which language laterality was investigated in a large group of healthy (*normal*) non-right-handers (e.g., Pujol et al., 1999; Knecht et al., 2000b; Szaflarski et al., 2002). In short, our results cannot be easily linked to any anatomical or functional abnormality, and most likely reflect a natural variation in the hemispheric specialization for language, which is usually underestimated (Kroliczak et al., 2011). The obtained outcomes are consistent with an earlier report which demonstrated that atypical language lateralization, which is a part of this continuum, is not related to impairments in intelligence, verbal fluency, or academic achievements (Knecht et al., 2001). Moreover, our study shows that this type of functional specialization is associated with quite specific neural characteristics, and connectivity profiles. Thus, both language systems yield similar behavioral outputs despite substantially different neural underpinnings. Below, we further discuss differences and similarities between the studied groups, both in terms of the disparate profiles of neural activity, and resting-state connectivity.

### Atypical lateralization of language: different or similar pattern of activity?

In general, when flipped across the x-axis, both of the studied groups exhibited highly similar pattern of activity during a verbal fluency task, yet with some notable differences. The commonalities and disparities between the sample with atypical language lateralization and our group of participants with typical language organization appear to correspond well to the two cerebral systems that could be engaged in the planning and execution of motor programs (Leiguarda and Marsden, 2000). The basal ganglia, and SMA, represent the system for overlearned skills (Grafton et al., 1995), with areas common for both groups and hemispheres. In sharp contrast, although IFG and PMv engagement is also common for both groups, it, nevertheless, clearly depends on hemispheric laterality. These two regions belong to the second system which is pronounced during the planning/execution phases of movement (Kroliczak et al., 2011; Vingerhoets et al., 2012), a system which has been also shown to mediate less rehearsed tasks (e.g., Kroliczak et al., 2007). This network is engaged noticeably more in the sample with atypical laterality as it includes also the right parietal and temporal cortices. In the group with typically organized language the processing is narrower and, at least for language tasks, is limited to greater signal modulation in the left IFG. This latter region, together with MFG and SMG, belongs to the praxis planning network (Johnson-Frey et al., 2005; Kroliczak et al., 2008; Przybylski and Kroliczak, 2017) characteristically engaged in typical populations and tasks (see also Marangon et al., 2016), and whose subdivisions are also typically invoked during action imitation (Kubiak and Kroliczak, 2016).

The substantial engagement of both the above-mentioned neuromotor mechanisms in a verbal fluency task is not that surprising. Indeed, recent evidence suggests the existence of a close link between the lateralization of language and praxis (Kroliczak et al., 2011; Vingerhoets et al., 2013; Goldenberg and Randerath, 2015; see also Kroliczak et al., 2016; Corballis, 2017). Specifically, individuals with atypical organization of language demonstrate also atypical representation of skilled movements (praxis). Here, we showed that atypical language laterality is related to widespread changes in the network potentially specialized for both language and praxis.

Importantly, in the sample with atypical laterality there were also differences in signal modulations that could not be explained by a mirror reversal of the left hemisphere activity. Specifically, the *atypical* sample engaged more the right ANG, middle part of the MTG, and SFG during the silent word generation task. These regions belong to the DMN (Greicius et al., 2003), which encompasses a large part of the frontal, temporal, and parietal cortices (Raichle, 2015). DMN is thought to support internal mentalization related to our plans for future, autobiographical memory recall, and other spontaneous thoughts not related to the task (Buckner et al., 2008). As this network is more active during rest (Binder et al., 1999), we did not observe its engagement in the contrast of our language task vs. resting baseline. Although there is no consensus among research concerning DMN detailed anatomy (see differences in the early formulation of the concept: Shulman et al., 1997; Binder et al., 1999; Mazoyer et al., 2001; Raichle et al., 2001), and possible existence of sub-networks (Sestieri et al., 2011; Braga and Buckner, 2017), the core elements of this system are ANG, posterior cingulate cortex, MTG, and SFG. Importantly, in two of the structures just mentioned, i.e., the ANG and MTG, neural activity was significantly greater in the right hemispheres of participants from the atypical group. In sharp contrast, in the typical group, the left ANG and MTG did not seem to be involved. As DMN is thought to be engaged in semantic processes (Binder et al., 2009), which are closely tied to language, some possible effects of language laterality on this network could not be excluded. Therefore, this result opens an exciting possibility of differences in the organization of this prominent brain network that are related to the lateralization of language. Indeed, these outcomes are also interesting in light of recent findings (Doucet et al., 2014) suggesting that the temporal lobe epilepsy, which could cause atypical laterality, alters the frontal parts of the DMN. Yet, our results clearly demonstrate that atypical language laterality in healthy individuals is related to a greater engagement of the two key posterior components of the DMN. This difference should then be of great clinical importance.

Overall, the widespread changes in the neural patterns of activity associated with atypical language lateralization involve substantial part of the classically-defined motor/praxis planning network in a “mirror fashion” to those with typical laterality. Arguably, there is also a much more important, and much greater engagement of the right hemispheric default mode components.

### Atypical lateralization of language: diffused or focused representation?

The nature of the hemispheric language representation is an important element of any theory of the lateralization of brain functions (Bishop, 2013). Some of these theories suggest that atypical language organization would entail a diffuse functional network (Brown and Hecaen, 1976). Our results partially corroborate this hypothesis, yet with an important caveat. We found that there is a strong negative relationship between the amount of cortical tissue used during the verbal fluency task and the absolute degree of lateralization. Specifically, only individuals with bilateral language representation demonstrated a more diffuse functional organization. Note that this relationship is non-trivial in the sense that different number of voxels (representing the extent of cortical tissue used) can give similar LIs (Seghier, 2008). In other words, bilateral language organization, represented with low LI scores, could be demonstrated by two small/focused clusters of highly symmetrical activity in BA 44/45, or a much more diffused—i.e., large in terms of spatial extent, even if with less symmetrical localization of foci of—activity in this ROI. Importantly, our analyses revealed that subjects with complete right hemisphere language lateralization demonstrate focused activation as individuals with typical laterality. As such, this outcome is similar to the result obtained in an earlier study (Knecht et al., 2003), which compared subjects with right hemisphere language lateralization with matched typical individuals. In the context of all these findings, it seems that there is a continuum of representations ranging from diffuse language network characterized by small absolute values of LIs, to more focal functional organization (either left or right lateralized) characterized by greater absolute values of LIs. These results fit well with an earlier proposal (Price and Crinion, 2005) that the dominant hemisphere for language inhibits the activity of the non-dominant one. Individuals with bilateral representation of language could lack of or have substantially smaller inhibitory influence of this kind. This could, in turn, result in a more diffuse language organization, as measured with the spatial extent.

The above-mentioned findings, based on voxel counting, may still require a dose of healthy skepticism. After all, some studies suggest that this method is suboptimal and has inherent drawbacks (Poldrack, 2007). Nevertheless, in the realm of language research, voxel counting is a reliable method which ensures sensitivity and specificity, even when compared to the Wada test, which is a standard in clinical practice (Dym et al., 2011).

### Peaks of activity: an interesting direction for future studies

The locations of peaks of activity within the Broca's area in both groups were different along the z-axis, with the one for the atypical group located lower. With this in mind, using the Neurosynth tool (www.neurosynth.org), we performed initial exploration of the connectivity differences that could be related to this result. Interestingly, we found that the mean peak of the typical group (*x* = −52, *y* = 14, *z* = 22) is widely connected with the WA and SMG, i.e., typical posterior language areas. In sharp contrast, the mean peak for the atypical group (*x* = 52, *y* = 14, *z* = 16) had only limited connectivity with the temporal lobe, and in the parietal lobe it was connected to the postcentral sulcus instead of SMG. Although, we are aware of the limitations related to the above results, we nevertheless point out that future studies should seriously consider such differences in connectivity patterns.

Three possible caveats related to these findings involve: substantial anatomical variability of the Broca's area (Keller et al., 2007), even small registration errors, as well as using a large smoothing kernel (here: of 6.2 mm). Indeed, it has been demonstrated that spatial smoothing can shift peaks of activity in the fMRI results (Jo et al., 2008). Nevertheless, we are convinced that the functional difference observed in this study between typical and atypical group could be of great scientific interest, particularly in the light of the above-mentioned alterations in connectivity. Indeed, studies using surface-based methods of registration and smoothing could easily validate our conclusions.

### Language laterality and handedness

An association between handedness and language laterality has been postulated almost from the very beginning of investigations on language representations in the brain. However, no clear relationships between these variables have been found (see Haberling and Corballis, 2015). Indeed, recent outcomes suggest that although there is some anatomical overlap between networks contributing to hand preference (handedness) and language laterality (i.e., right PMv) there is little functional overlap. Specifically, handedness can affect language laterality only indirectly, e.g., by influencing the praxis network (Gainotti, 2015; Haberling and Corballis, 2015; Badzakova-Trajkov et al., 2016), which is in turn more closely related to language (Corballis, 2003; Kroliczak et al., 2011, 2016; Vingerhoets et al., 2013). These results correspond well with recent studies suggesting that handedness and language lateralization are related only indirectly, but left-handedness increases the likelihood of bilateral or right-hemispheric language specialization (Somers et al., 2015; Joliot et al., 2016). Therefore, a right-handed individual with left hemisphere dominance would exhibit a similar functional organization as a left-hander with the left hemispheric specialization. Indeed, when we compared left-handers with right-handers and controlled for LIs no significant differences between these groups were found.

### Local resting-state connectivity differences

We found that the connectivity of cerebellum differs between the studied groups. Specifically, we showed that the left cerebellum in the group with typical language organization exhibited greater connectivity with the right early visual cortex. This result is consistent with recent observations that the cerebellum also plays a critical role in language processing (e.g., Booth et al., 2007), and language-related experience itself can influence the functioning of cortical networks for vision (Dehaene et al., 2010; Szwed et al., 2012; see also Siuda-Krzywicka et al., 2016). Yet, other studies which examined connectivity of the cerebellum did not find a link between these structures (Buckner et al., 2011). However, most of them used global signal regression that potentially alters the intrinsic connectivity. Although, the exact functional importance of the link between the cerebellum and early visual cortices can be debated, our results clearly show that this pattern of connectivity is influenced by language lateralization.

### Global resting-state connectivity differences

Somewhat surprisingly, the most robust results were found when we examined differences in the hemispheric resting-state global signals between groups (i.e., at a global but not local connectivity level). Specifically, both groups differ in the connectivity of the ventral attention and DMNs. Recently, hemispheric global signal from the left hemisphere has been mapped onto language related areas, whereas right hemispheric global signal has been linked to the attention network (McAvoy et al., 2016). Our results corroborate these findings, at the same time substantially extending their interpretation. Specifically, we have demonstrated that atypical language laterality can alter even the hemispheric global signal during resting-state. These results correspond well with the outcomes from a recent study which showed that there is a complementary hemispheric specialization for language and visuospatial attention (Cai et al., 2013). In fact, as our results suggest, this complementary specialization is also reflected in the hemispheric global signal. Moreover, we found that the asymmetry of hemispheric global signal at rest affects the laterality of the DMN. This finding parallels well with the outcomes from our language task, which showed that atypical individuals utilized more the right hemisphere DMN components during silent word generation.

### Clinical importance

Our results indicate that a transfer of language functions from one hemisphere to another is associated with widespread alterations in connectivity and often a more diffuse representation of language itself. This complex process could be influenced by a variety of variables, e.g., the age at which an epileptic episode occurs in the left hemisphere, the extent of a lesion, structural asymmetries of unknown etiology, etc., and, therefore, could result in diverse outcomes. Indeed, recent studies (Liegeois et al., 2004; Raja Beharelle et al., 2010) suggested that in some cases the right hemisphere may not be capable of sub-serving language functions in the face of an early left brain injury. Therefore, our results showing alterations related to the atypical language laterality in a healthy brain are of vital importance for the clinical practice by showing changes that possibly must occur in the injured brain to fully accommodate language functions. Indeed, a recent study (Yourganov et al., 2016) showed that this approach, utilizing mainly connectivity data, could predict post-stroke language impairments.

### Generalizability of the obtained results to representations of other languages

Although native speakers of Polish (the most commonly spoken Western Slavic language) were tested in this project, the outcomes should be easily generalizable to other languages, including English. Of course, when compared to modern English, Polish has some unique features: rich inflectional morphology, grammatical gender, relatively free word order, as well as some differences in phonology to name just a few. Yet, in earlier studies from our laboratory we convincingly demonstrated that the lateralization of single word utterances and processing is quite similar in Polish and English (Krefta et al., 2015; Klichowski and Kroliczak, 2017).

## Conclusions

The fact that more than one neural mechanism can give similar output seems to be still underappreciated in cognitive neurosciences. Here, we showed that atypical language lateralization is a part of a natural continuum of hemispheric specializations. This type of functional representation seems to be related to handedness, yet only in an indirect way, i.e., it has some anatomical overlap but little functional connection. If it is bilateral it then entails a more diffuse representation of language functions. Moreover, individuals with atypical language organization engage more the right DMN components during a language task. There are also important differences in neuronal responses that manifest themselves during resting-state. Specifically, right-sided and bilateral representation of language alters brain connectivity of the cerebellum, and even leads to changes in the hemispheric resting-state global signal. Importantly, these differences are not accompanied by any vivid behavioral impairment, or brain abnormality. Therefore, we conclude that atypical lateralization of language is a natural and unique variant of functional representation.

## Author contributions

This project was designed by SB and GK. Data were collected by ŁP and MP, analyzed by SB, and interpreted by SB, MP, ŁP, and GK. The manuscript was written by SB and GK.

### Conflict of interest statement

The authors declare that the research was conducted in the absence of any commercial or financial relationships that could be construed as a potential conflict of interest.
